# Spectroscopic Investigation of Local Mechanical Impedance of Living Cells

**DOI:** 10.1371/journal.pone.0101687

**Published:** 2014-07-07

**Authors:** Luca Costa, Mario S. Rodrigues, Núria Benseny-Cases, Véronique Mayeux, Joël Chevrier, Fabio Comin

**Affiliations:** 1 European Synchrotron Radiation Facility, Grenoble, France; 2 Université Joseph Fourier, Grenoble, France; 3 Centro de Física da Matéria Condensada/Dep. Fisica, Faculdade de Ciência, Universidade de Lisboa, Lisboa, Portugal; 4 Astbury Centre for Structural Molecular Biology, Leeds University, Leeds, United Kingdom; 5 Centre National de la Recherche Scientifique, Institut NÉEL, Grenoble, France; 6 Université Grenoble Alpes, Institut NÉEL, Grenoble, France; Tufts University, United States of America

## Abstract

We studied nanoscale mechanical properties of PC12 living cells with a Force Feedback Microscope using two experimental approaches. The first one consists in measuring the local mechanical impedance of the cell membrane while simultaneously mapping the cell morphology at constant force. As the interaction force is increased, we observe the appearance of the sub-membrane cytoskeleton. We compare our findings with the outcome of other techniques. The second experimental approach consists in a spectroscopic investigation of the cell while varying the tip indentation into the membrane and consequently the applied force. At variance with conventional dynamic Atomic Force Microscopy techniques, here it is not mandatory to work at the first oscillation eigenmode of the cantilever: the excitation frequency of the tip can be chosen arbitrary leading then to new spectroscopic AFM techniques. We found in this way that the mechanical response of the PC12 cell membrane is found to be frequency dependent in the 1 kHz - 10 kHz range. In particular, we observe that the damping coefficient consistently decreases when the excitation frequency is increased.

## Introduction

Atomic Force Microscopes (AFMs) are intensively used in the studies of cells. By mapping the morphology at the nanoscale, AFMs have been largely employed for imaging the cell surface and the submembrane cytoskeleton [1]–[3]. Nowadays, AFMs are also used in molecular recognition experiments and in the exploration of the energy landscape of receptor-ligand interactions in living cells [4], [5]. An interesting application is given by the possibility of applying a force to the cell membrane and measuring the cell elasticity: the mechanical response of the cells is a key observable for diseases diagnostics [6] and cell signalling [7], [8]. More generally, the elasticity is involved in many of the physiological processes performed by the cell. Due to the cell viscoelastic behavior [9], [10], the observed mechanical properties could change significantly depending on the frequency probed during an experiment. In this frame we focus on the measurement of the mechanical impedance of PC12 living cells employing atomic force microscopy methods.

In conventional static AFMs, the tip is slowly put in contact with the cell membrane for recording a force vs indentation curve. Depending on the geometry and the nature of the mechanical contact between the tip and cell, different contact models can be invoked to extract then the intrinsic elasticity of the cells [11], [12]. The determination is based on statistics over a large number of curves in order to properly quantify the cell Young modulus. The procedure may be applied on an array of different locations on the cell to get a two-dimensional distribution of the Young modulus [13], [14]. This technique is often time-consuming because of the large number of force curves needed to be acquired. Recently, advanced AFM operational schemes have been proposed [15], [16] for measuring nanomechanical properties of soft samples. These methods, called multifrequency AFM, allow the user to simultaneously acquire the topography and the mechanical properties of the specimens. This can be done either monitoring the behavior of higher harmonics excited while the tip interacts with the sample [16], either by direct excitation and monitoring of the 2*^nd^* cantilever eigenmode [17]. The Multifrequency techniques are much faster than conventional force mapping. However, in both cases the sample elasticity is probed at frequencies related to the cantilever eigenmodes.

We developed a different type of AFM instrument based on fiber optic detection system that we called Force Feedback Microscope (FFM) [18], [19]. This instrument allows the user to simultaneously measure the static force, the elastic force gradient and the damping coefficient, fully characterizing the interaction between the AFM probe and the specimen. The static force is the output of an active feedback loop that controls the average position of the tip in space. A sub-nanometric oscillation amplitude is then imposed to the tip for measuring the force gradient and the damping coefficient. The use of small oscillation amplitudes intrinsically implies that the Force Feedback Microscope operates in a linear regime. Moreover, the small amplitude of oscillation and the possibility of using at the solid/liquid interface very soft cantilevers (0.01 N/m) minimizes the invasiveness of AFM experiments [20] on soft samples. A key feature of the Force Feedback Microscope is the possibility of using as feedback signal to record the sample morphology either the force, the force gradient, or the damping coefficient. One out of these three different quantities can then be arbitrarily kept constant, providing a contrast in the other two physical observables. This contrast then is intrinsically dependent on the choice of the feedback signal, the magnitude of the setpoint, the nature of the interaction and the local change of the interaction on specimens [19]. An important feature of the FFM is the possibility to arbitrarily choose the excitation frequency imposed to the cantilever. This capability is used in this paper to characterize the mechanical response of PC12 living cells in the 1 kHz - 10 kHz frequency range. For this purpose, PC12 living cells have been previously characterized in conventional static mode. Subsequently, cells have been imaged at different constant forces in the FFM mode. The topography is acquired simultaneously to the measured sample stiffness and the damping coefficient. Finally, a set of indentation curves at different excitation frequencies has been recorded at the surface of the PC12 living cells to quantify the mechanical response in the 1–10 kHz range.

## Materials and Methods

### Cell line

PC12 were obtained from ATCC. PC12 is a cell line derived from a pheochromocytoma of the rat adrenal medulla and is extensively used as a neuronal model. In the experimental conditions used the cells were not differentiated.

### Cell culture

Some existing protocols have been followed [21], [22]. The PC12 cells are cultivated inside 250 ml Flasks produced by *BD Falcon*. The cell medium is a solution of Minimum Essential Medium, with 10% Fetal Bovine Serum, supplemented with antibiotics 1% Penicillin-Streptomycin at 100 ng/ml. All the products were produced by *Life technologies*. The medium is changed every three days. Cells are cultivated inside an incubator at 37°C, 5 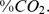


### Glass support for AFM measurements

*Glass coverslips* (Thermanox, 13 mm diameter) are previously autoclaved for sterilization. The coverslips are deposited inside steryle 4 wells Culture plates (*Thermo Scientific Nunc dishes IVF*). 500 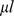 of Fibronectin 1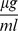 (*Life technologies*) are deposited in each culture plate and the sample is then left incubated for 60 minutes at room temperature. The Fibronectin is then removed and each culture plate is washed 4 times with sterile PBS.

500 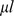 of MEM with 10% Fetal Bovine Serum and 1% Penicillin-Streptomycin are deposited in each culture plate.

### Cells transfer on the glass support for the AFM measurements

The buffer inside the flask is removed. A solution of 15 ml PBS, 0.25% Trypsin at 1 mg/ml is transferred inside the flask. The flask is then left incubated for 30 minutes at 37°C. The cells are now suspended in the solution. 500 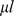 of fetal bovine serum are added to the solution. The solution is centrifuged for 5 minutes at a relative centrifugal force of 22340 g inside a 15 ml centrifuge tube. The centrifuge is an Eppendorf 5702R with a rotational radius of 12.8 cm. The solution is removed leaving the cells at the bottom of the tube. 2 ml of MEM, 10% fetal bovine serum, 1% Penicillin-Streptomycin at 100 ng/ml are inserted in the tube. Cells are mechanically re-suspended using a Pasteur pipette. The cells concentration is then calculated using a *Neubauer cell*. Cells have been usually deposited in each culture plate on the top of a coverslip glass at a concentration of 1 million/ml. The cells are then left in the incubator at 37°C, 5 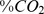 for one/two nights and then measured with the FFM. The imaging buffer is MEM, 1% Penicillin-Streptomycin at 100 ng/ml.

### Force Feedback Microscopy

The Force Feedback Microscope is a custom - made AFM based on a fiber-optic interferometer detection system [18]. Triangular and rectangular MLCT cantilevers provided by *Bruker* with nominal spring constants of 0.01 N/m and 0.02 N/m respectively have been employed. Both the cantilevers have a nominal tip radius of 20 nm. When damped in liquid and measured with an optical fiber, their resonance shifts down to less than 3 kHz. The proximity of the fiber to the the cantilever increases the broadening of the resonance and can effectively suppress it.

An *Asylum Research* fluid cell has been modified to fit properly in the home made FFM. The glass coverslips have been fixed inside the liquid cell. Cells have been kept at 37°C by regulating the temperature of the imaging buffer during the measurements. A central aspect of the FFM is the calibration of the instrument. The instrument is calibrated by recording a set of approach curves between the tip and the glass for each excitation frequency 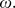


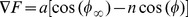
(1)

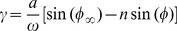
(2)


Equations (1) and (2) convert the measured values 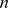 and 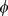 into the interaction properties 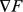 and 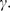 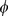 is the phase between the oscillation of the tip and excitation imposed at the cantilever base, whereas 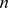 is the so-called normalized force/response ratio. 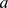 and 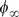 are parameters dependent on the cantilever employed and the experimental environment (gas, liquid, vacuum). Equations (1) and (2) are derived in reference [23]. To be noted that these equations can be used mainly because the oscillation amplitude imposed to the tip is kept small (below 1 nm) implying that the FFM can be considered as a linear AFM.

The cantilever is mechanically excited at its cantilever base. Recent works indicate that in optical beam deflection setups the displacement of the cantilever base in liquid is not negligible and suggest more accurate methods to quantify the interaction [24], [25]. Since the FFM measures the *absolute* tip position with a fiber optic, the displacement of the cantilever base does not influence the calibration and the measurements.

**Figure 1:**
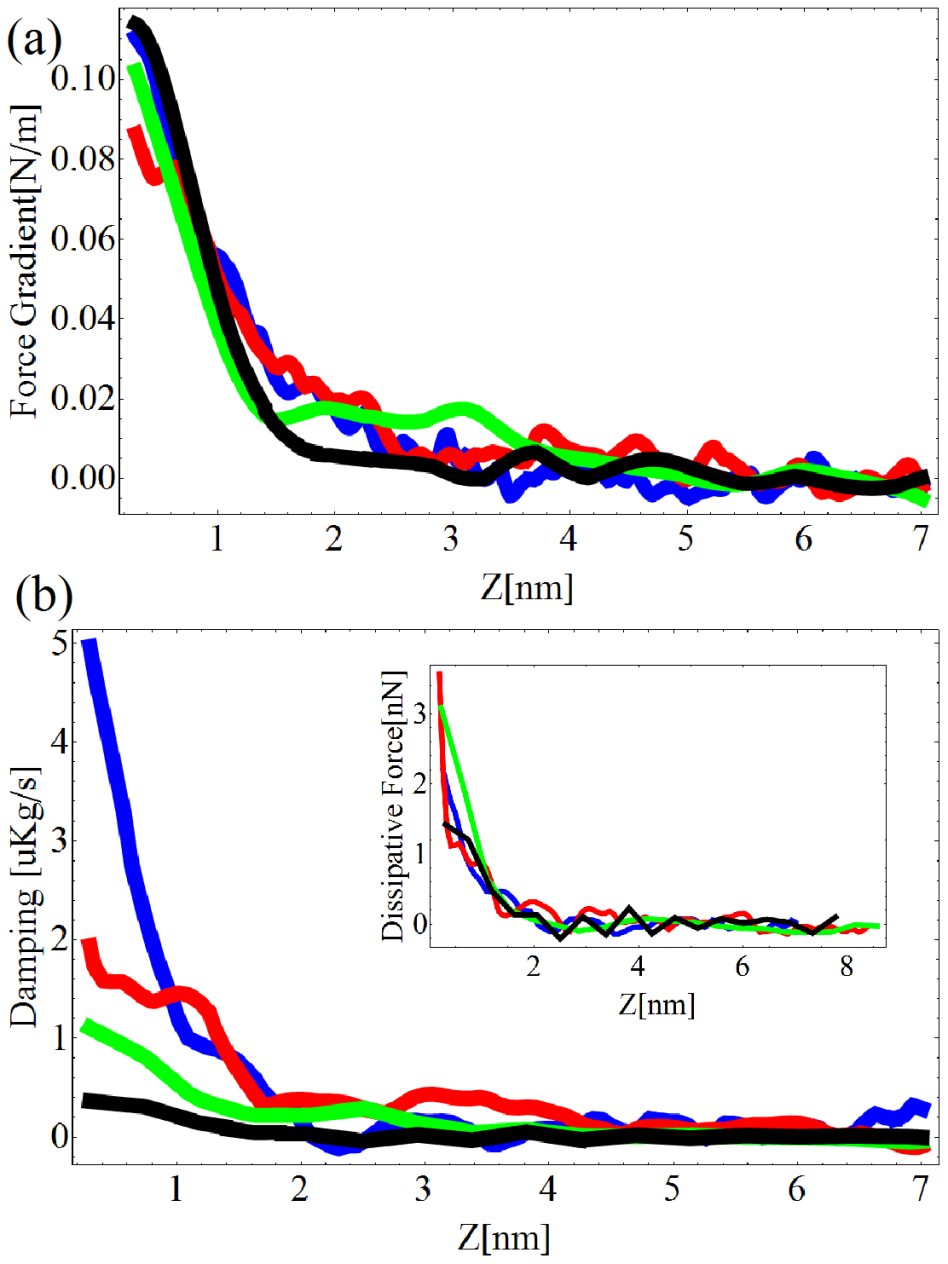
Spectroscopy of the glass in liquid buffer. Force gradient (a) and damping factor 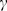 (b) as a function of the excitation frequency. Excitation frequencies: Blue  = 1.11 kHz, Red  = 3.11 kHz, Green  = 9.11 kHz, Black  = 11.11 kHz.

Assuming that the integral of the force gradient is equal to the measurement of the static force between the AFM probe and the glass, the key point is to determine the parameters 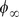 and 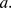 The protocol of calibration is explained in details in reference [18]. Figure 1 shows the elastic force gradient and the damping coefficient measured on the glass for four different excitation frequencies. Each curve is averaged over three different runs. The elastic force gradient is found to be independent of the frequency probed (figure 1a). The damping coefficient is observed to decrease at larger excitation frequencies (figure 1b). Since the dissipative force is proportional to the damping coefficient times the speed of the tip, we do not observe a significant decrease in the force needed to maintain the tip oscillations constant when the excitation frequency is increased. In other words, the dissipative force exerted by the glass on the AFM tip is found to be constant in the 1 kHz–10 kHz range.

One question may arise concerning the validity of the calibration performed on the glass, once the tip is on the top of living cells. The long-range hydrodynamic squeeze film damping between the oscillating cantilever and the cell surface may in fact be different depending if the tip is on the top of the cell or on top of the substrate. Most of the measurements shown in this manuscript have been performed on substrates completely covered with cells. The calibration was then performed in the small portion of the sample where the glass was not covered by the cells. Since the long-range hydrodynamic squeeze film affects the cantilever motion, both the measurements on the glass and the measurements on the cells are equally affected by this effect, which is taken into account during the calibration procedure.

Another question that may arise is the influence of spurious resonances in the cantilever transfer function in liquids on the evaluation of 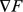 and 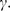 The issue is well known and studied in the supplementary section of reference [25] for measuring stiffness and dissipation in the small oscillation amplitude regime for optical beam deflection operational schemes. Spurious resonances may couple to the cantilever base or directly to the tip, in which case the effect is usually called *fluid-borne excitation* [25]. It can be shown theoretically that the effects of both spurious resonances is directly taken into account in equations (1) and (2) through the calibration procedure of the parameters 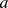 and 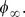 In addition, we compared experimentally force gradients acquired in FFM mode with a conventional dither acoustic excitation with force gradients acquired in FFM mode with a direct capacitive excitation of the tip. We observed a difference in the calibration parameters 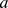 and 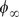 linked to the different actuation scheme. Both the schemes provide however the same results in terms of conservative and dissipative interaction [26].

In conclusion, the measurement of the tip position leads to many advantages compared to the optical beam deflection scheme. When measuring in liquids, the cantilever base displacement does not influence the measurements and the effect of the spurious resonances in the cantilever transfer function is taken into account by the calibration procedure of the cantilever dynamics described in this section.

## Results and Discussion

### Characterization in conventional static mode

**Figure 2:**
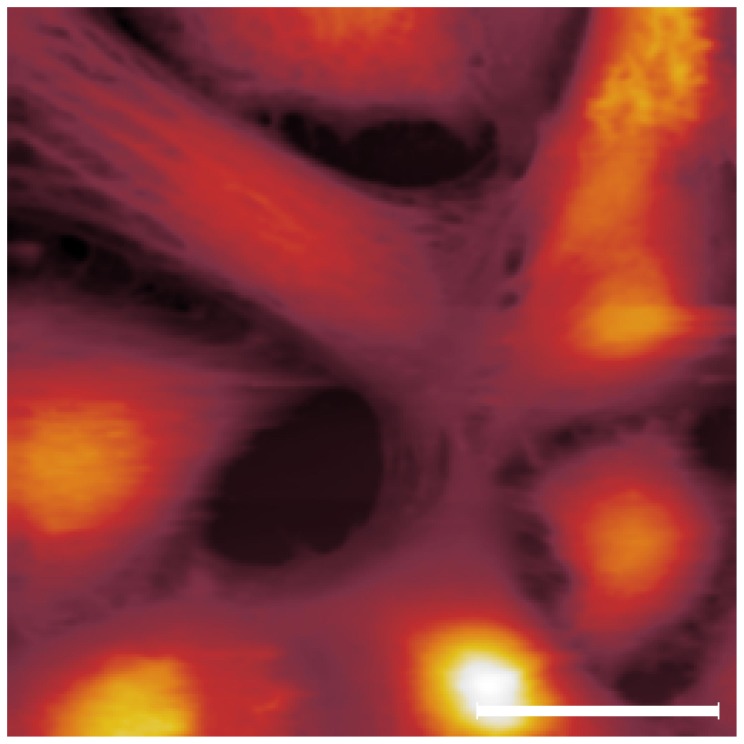
PC12 living cells imaged in conventional contact mode. Scale bar  = 30 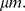 Several cells of different shapes are here imaged.

Cells have been firstly characterized in conventional AFM static mode. Figure 2 shows a typical image acquired at a constant repulsive force of 100 pN. Since in our custom - made AFM it is not possible to laterally offset the sample from the tip, a scan area of 100×100 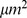 is the only sample surface accessible to obtain an image. As a consequence, the cell concentration employed for the cell culture has been refined in order to obtain a cell density on the glass support similar to the one presented in the image. Indeed, the presence of the glass support in the scan area is a necessary condition for a good calibration of the FFM.

Once successfully imaged, the living cells have been indented to extract a set of force curves. Modeling the tip as a cone indenter, the relationship between the force 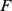 and the indentation 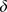 [10], [27] can be given by
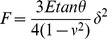
(3)where 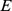 is the Young modulus and 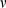 is the Poisson ratio of the cell. 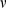 is assumed to be equal to 0.5; 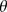 is the cone semi-angle. Since the MLCT probe is a four-sided pyramidal indenter, we treated it as a cone indenter with an effective semi-angle 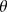 of 22.9°

**Figure 3:**
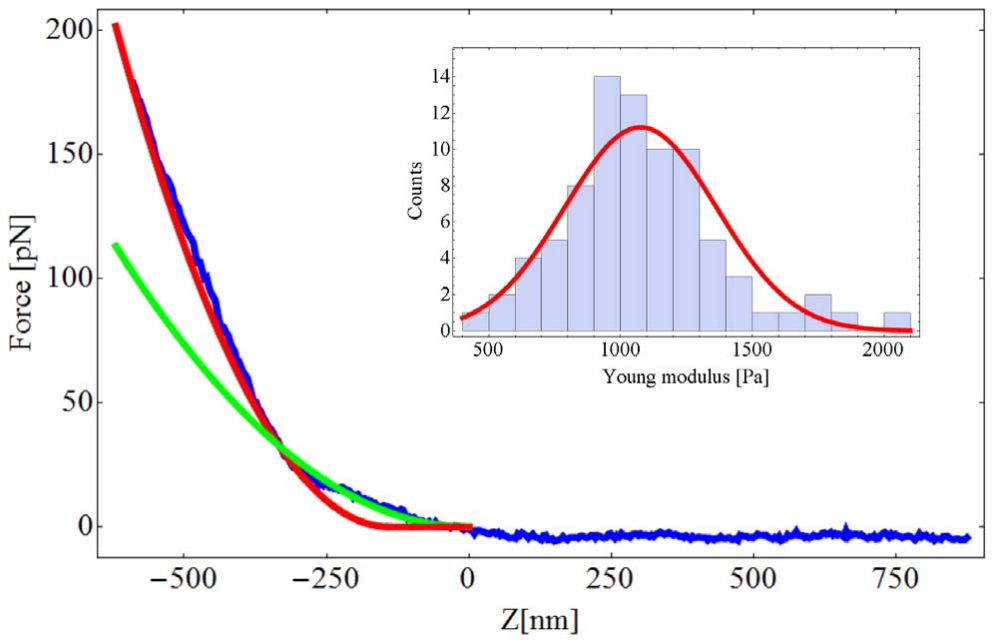
Indentation static curve on a PC12 (blue) fitted with equation (3) (red). The fit of the soft outer layer of the cell, referred as “brush layer” in the text, is in green. The tip is modeled as a four-sided pyramidal indenter. The Young modulus extracted is 1070 Pa using a Poisson ratio equal to 0.5 and 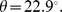 Inset: Histogram of the distributions of the PC12 Young's modulus. The quasi-static value is 1.1 kPa±0.3 kPa.

Equation (3) has been employed to evaluate the static Young modulus of the living cells. However, the assumption that the Young modulus does not depend on the indentation depth does not apply to heterogeneous media like cells. At first, if the indentation depth is too high, the glass substrate may affect the measurement, causing an overestimation of the Young's modulus. The maximum indentation depth reached in our experiments is 600 nm, which is consistently lower than the height of the PC12 cells (3–5 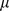
m). Then, an underestimation of the extracted Young's modulus may be caused by inhomogeneities at the cell surface. The inhomogeneities induce an indentation depth dependence of the Young's modulus which may result in wrong estimations when using the model introduced in equation (3). Figure 3 shows an indentation experiment where this situation occurs. Clearly, the probe is pushing through two different layers. A softer layer corresponding to the cell surface elasticity which is fitted by the green curve and shows a Young's modulus of 450 Pa. A second, stiffer layer is fitted by the red curve which shows that the elasticity of the cytoplasm is 1070 Pa. The softer outer layer may be referred to the so called “brush layer” which is important for the cell to interact with the external environment [28], [29]. It is therefore of importance to properly choose the right part of the indentation curve to be fitted when using the model described in equation (3). A statistic over 81 force curves taken on a same cell is shown in the inset of Figure 3. In this work, we have taken into account the elasticity of the cytoplasm which has been evaluated with equation (3) for large indentation depths. The static Young modulus is 1.1 kPa±0.3 kPa, which is comparable to the one measured on PC12 living cells in previous works [30]. The supplementary section includes 9 additional force vs indentation curves that have been used in the statistical evaluation of the PC12 Young's modulus.

### Characterization at constant force in Force Feedback Mode

**Figure 4:**
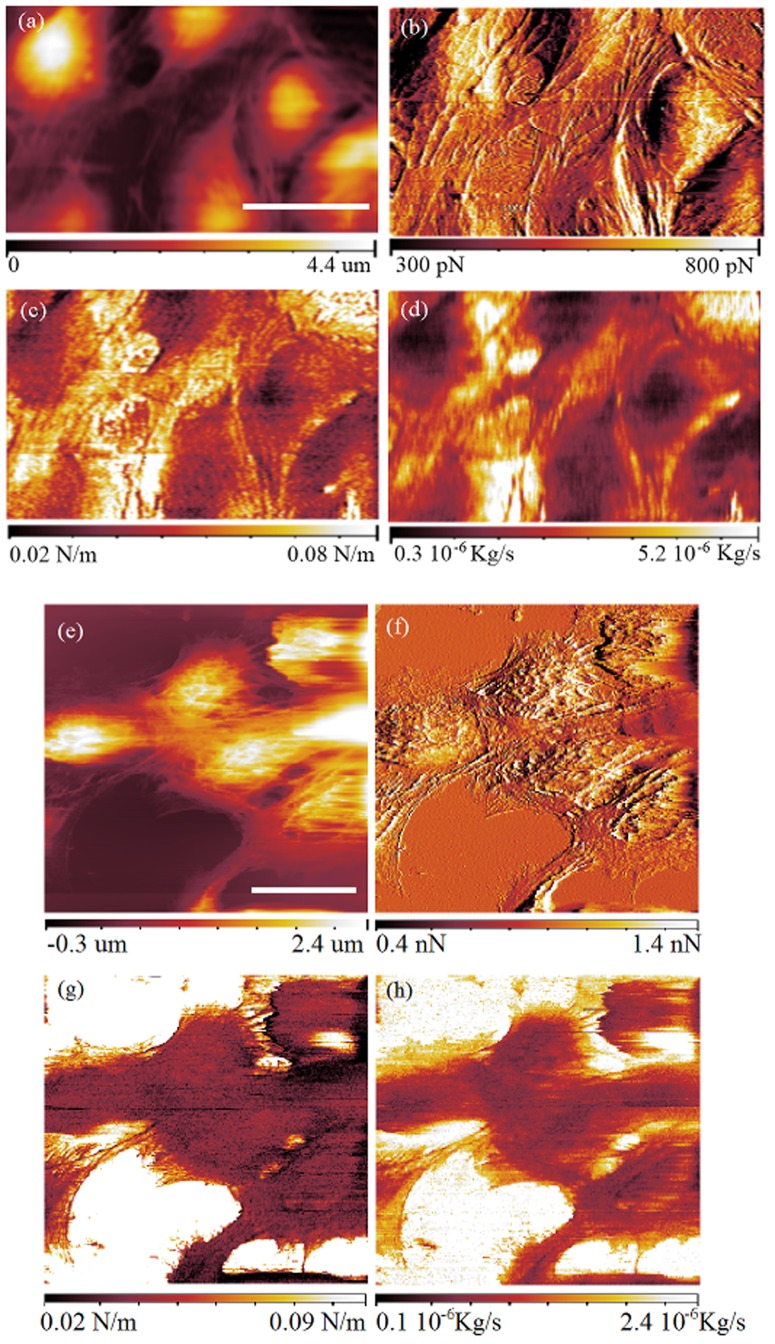
FFM images of PC12 living cells acquired at a constant force of 500 pN and 1 nN. In the images at 500 pN constant force (a,b,c,d) the scale bar is 30 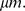 a) Sample topography, b) force between the tip and the sample (error), c) force gradient and d) damping factor. In the images at 1 nN (e,f,g,h), the scale bar (a,e) is 30 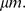 e) Sample topography, f) force (error), g) force gradient and h) damping factor.

Cells have been imaged in FFM mode at a constant repulsive force of 500 pN. In this force constant mode, the topography is acquired simultaneously to the force gradient and the damping factor. An amplitude of 0.4 nm has been imposed to the tip at 2.2 kHz. Figure 4a shows the topography, while figure 4b shows the force between the tip and the sample which is the error signal for this mode. Figure 4c is the measurement of the force gradient and figure 4d is the damping factor.

The membrane is found to be softer in the center of the cell than at the periphery. In analogy with the stiffness, the damping factor is observed to be lower in the center of the cell than at its borders. In fibroblasts, these effects can be due to the conformation of the cytoskeleton, in particular of the actin filaments and the microtubules inside the cell as suggested by [14], [31], [32]. These effects can also be due to the higher influence of the glass substrate when the measurements of the stiffness and the damping factor are carried out at the periphery of the cells. The measured stiffness of the cells is comparable to the stiffness measured in multi-harmonic atomic force microscopy on different cells [16].

In a different experiment, PC12 cells have been imaged at the constant force of 1 nN. An amplitude of 0.4 nm was imposed to the tip at 7.78 kHz. Figure 4e shows the topography, figure 4f the force (the error signal), figure 4g the elasticity and figure 4h the damping coefficient.

Figure 4e reveals the presence of actin filaments/microtubules which are however hardly visible in the elasticity and damping coefficient images. In analogy with the measurement at constant force of 500 pN, stiffness and damping factors are observed to be lower in the center of the cells than at the periphery of them.

Although the cytoskeleton starts to be visible at higher applied force (figure 4a and figure 4e), we were not able to characterize mechanically the cytoskeleton because of the very small oscillation amplitudes that we use and the consequent loss in sensitivity.

### Characterization at constant force and variable frequency

**Figure 5:**
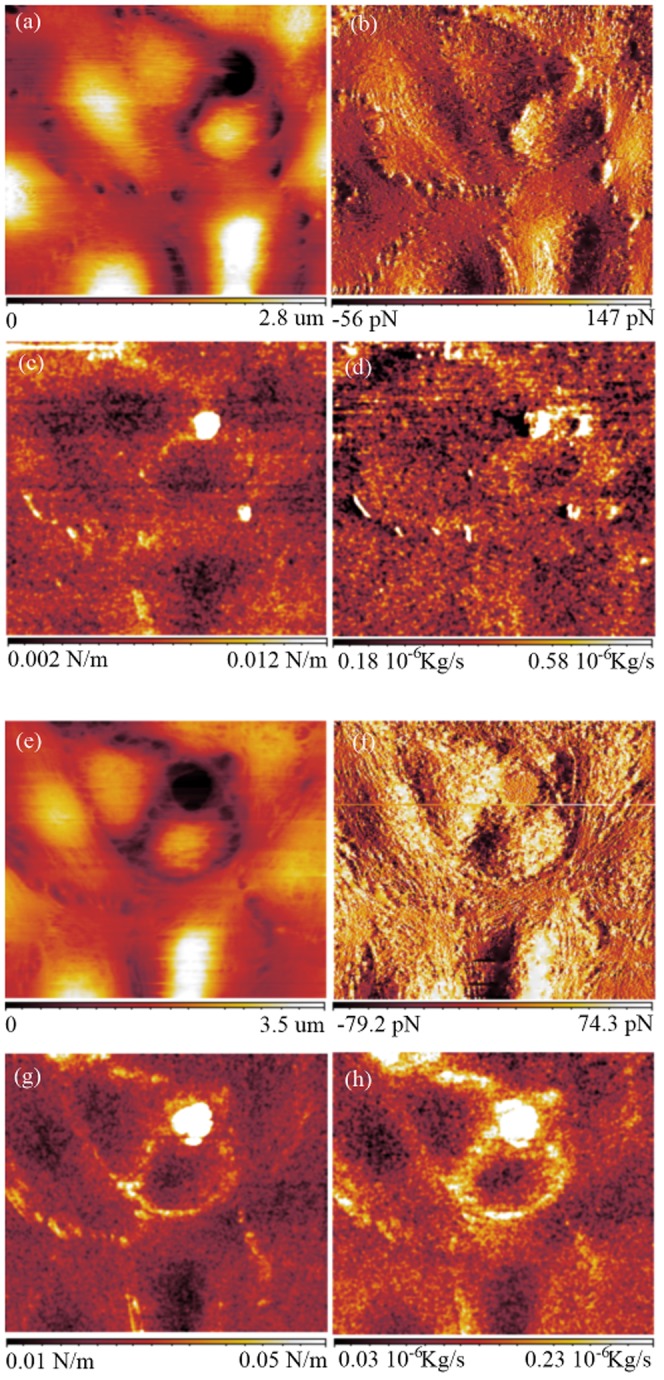
FFM images of PC12 living cells acquired at a constant force of 50 pN. Scan area  = 90×90 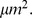 a) Sample topography, b) force between the tip and the sample (error), c) force gradient and d) damping factor at 2.25 kHz. e) Sample topography, f) force (error), g) force gradient and h) damping factor at 13.25 kHz.

The freedom that the FFM leaves in the choice of the excitation frequency of the AFM tip allows the user to measure the sample force gradient and the sample damping factor at any arbitrary frequency. To profit of this opportunity, a set of calibration curves on the top of the glass substrate were acquired for 2.25 kHz and then for 13.25 kHz with an oscillation amplitude of 0.4 nm. Once the FFM calibrated, images at constant repulsive force of 50 pN were acquired at those excitation frequencies (figure 5a,b,c,d for 2.25 kHz and 5e,f,g,h for 13.25 kHz). In analogy with the measurement presented in figure 4, the cell center is again observed to be softer than the cell borders.

The stiffness and the damping factor measured at 2.25 kHz are nosier than those measured at 13.25 kHz mainly because of the larger influence of the noise 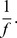 In order to get an acceptable measurement at low frequency, the scan speed at 2.25 kHz has been set twice slower than the one at 13.25 kHz.

The changes in the sample morphology between figure 5a and figure 5e are due to the fact that the cells are alive. We observe consistent differences between the images of the elasticity and the damping factor. The cells are found to be stiffer at 13.25 kHz than at 2.25 kHz by a factor five. The damping factor is instead observed to be larger at 2.25 kHz than at 13.25 kHz. The decrease of the damping factor for higher excitation frequencies is a behavior observed already on the spectroscopy performed on the glass (figure 1).

### Indentation curves

A spectroscopic investigation of the PC12 cells has been carried out through the acquisition of tip-cell approach curves with the following protocol:

- The cells were imaged in conventional contact mode.
- The FFM was calibrated with a set of approach curves at different frequencies on the glass, providing a set of parameters 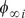 and 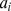 for each excitation frequency 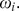 The force gradients 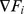 are the same for all excitation frequencies as shown in figure 1.
- A set of approach curves at different frequency on the PC12 was performed in the highest point of the cell topography.

**Figure 6:**
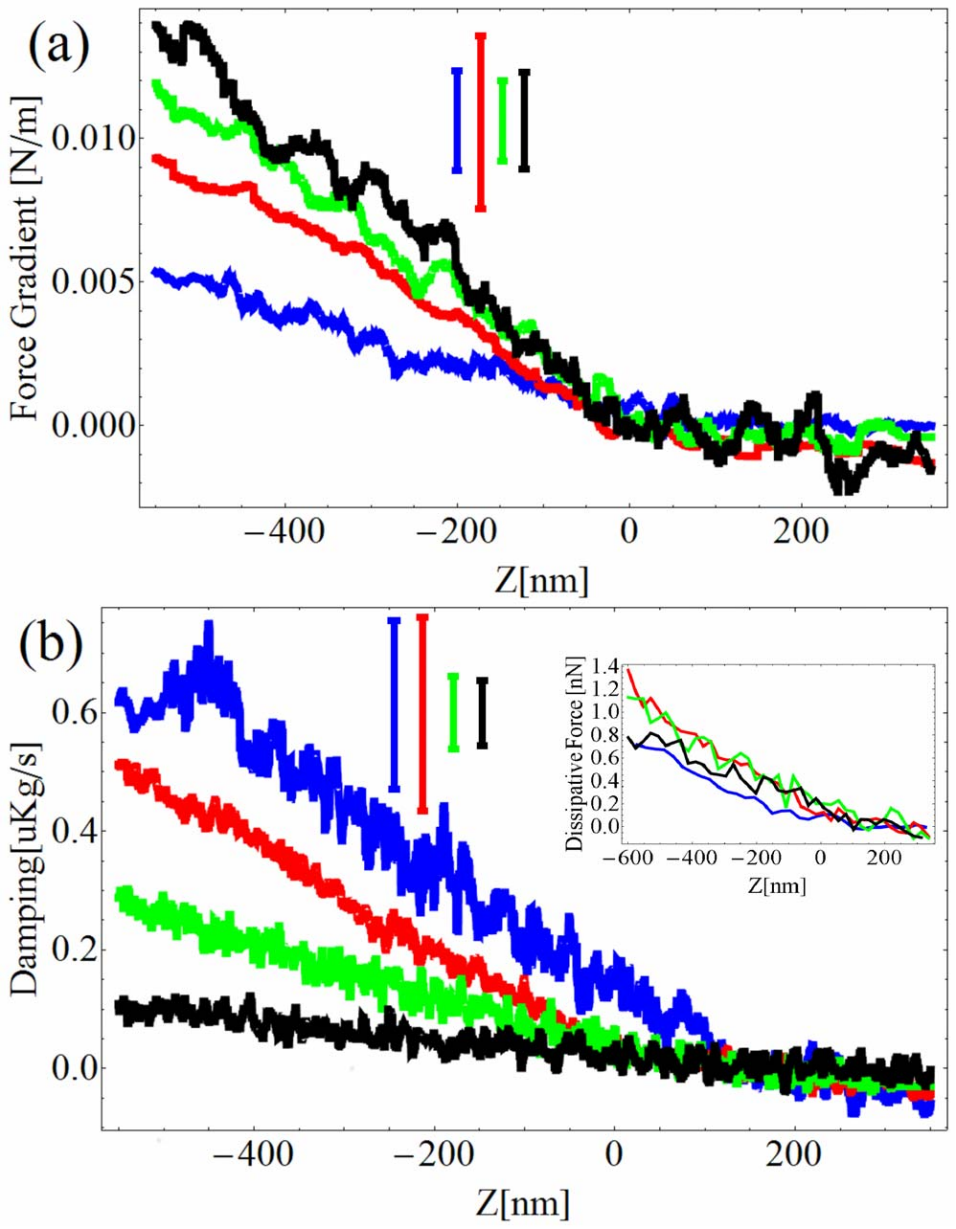
Spectroscopy of the PC12 in liquid buffer. Force gradient (a), damping factor (b) and dissipative force (inset) as a function of the excitation frequency. Excitation frequencies: Blue  = 1.13 kHz, Red  = 5.13 kHz, Green  = 7.13 kHz, Black  = 11.13 kHz. The error bars are shifted upward for clarity.

For an applied force of 500 pN, the observed cell elasticity in all the measurements has always been found in the order of 0.01 N/m. An example of such an experiment is presented in figure 6. The force gradient and the damping factor averaged over five approach curves are reported for four different excitation frequencies.

We note that the damping rate is lower when the excitation frequency increases, whereas the force gradient is increasing with the frequency. The increase of the elasticity with the frequency is not consistent in all the performed measurements. The decrease of the damping factor with the frequency is, on the contrary, reproducible. The resulting dissipative force, presented in the inset of figure 6b, is observed to be poorly dependent on the frequency. According to [10], the damping factor increases with the excitation frequency in the 1 Hz–100 Hz range. However, in our case the oscillation amplitude imposed to the tip is more than two orders of magnitude lower than the usual amplitudes used for studying living cells. Since in the present case the oscillation amplitude is much smaller than the cell membrane thickness, we think that the contribution of what is below the membrane may be smaller when compared to the case where the amplitude of oscillation is larger than the cell membrane thickness. In addition, a small oscillation amplitude may induce measurements of stiffness and damping factor which are more localized on the cell membrane than in an experiment where larger oscillation amplitudes are imposed to the tip.

### Analysis of elastic properties in dynamic mode

Focusing on the elasticity, from equation (3) we can define the force gradient as a function of the tip indentation as:
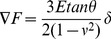
(4)


**Figure 7:**
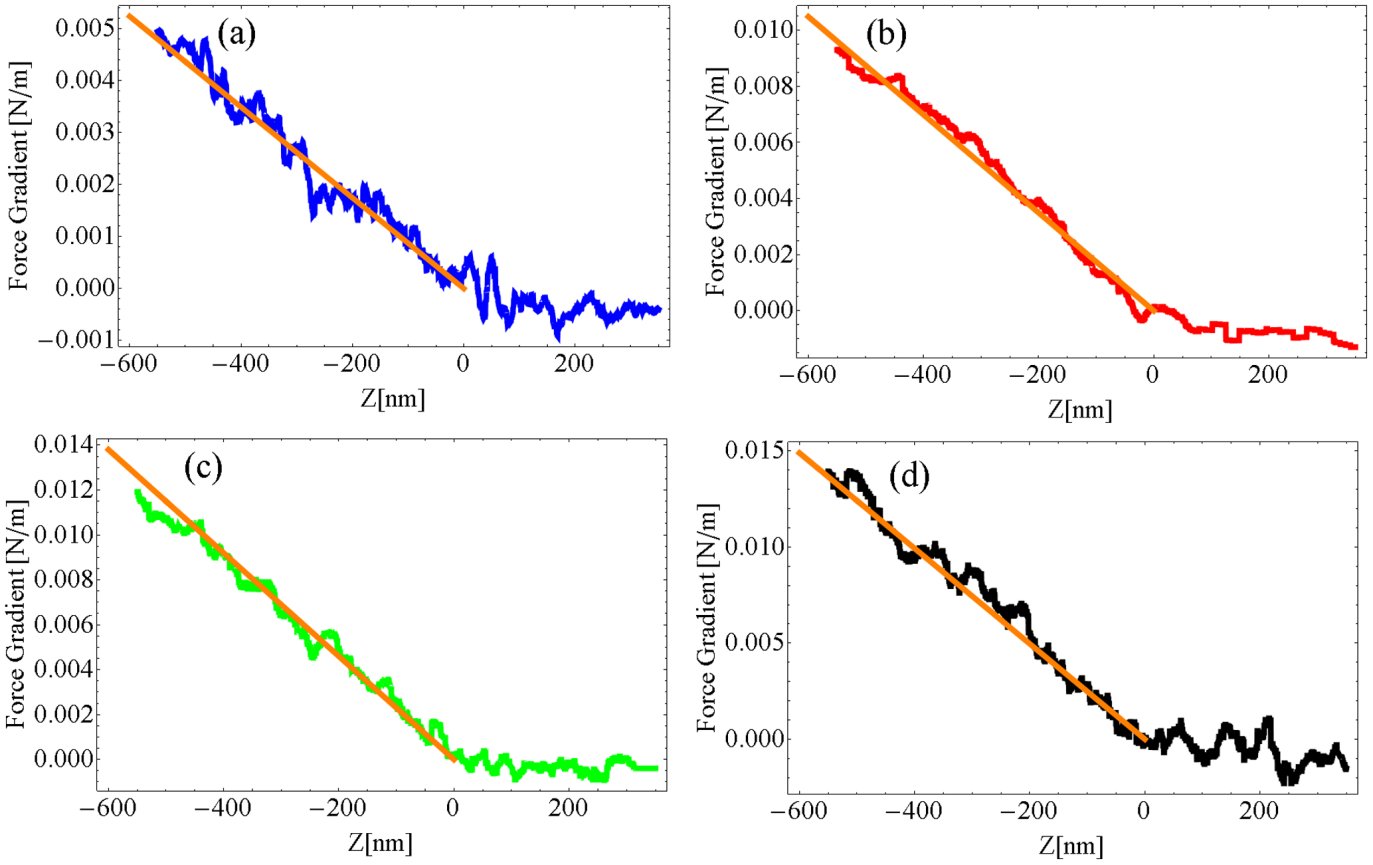
Analysis of the PC12 elastic properties through indentations curves. a) Force gradient as a function of the tip indentation. a) Excitation frequency  = 1.13 kHz, b) Excitation frequency  = 5.13 kHz, c) Excitation frequency  = 7.13 kHz, d) Excitation frequency  = 11.13 kHz. The lines in orange are the experimental linear fit of the cell elasticity using equation (4).

In particular, the data presented in figure 6a have been used to extract the cell Young modulus as a function of the excitation frequency. The Young modula are found to be equal to (10±3) kPa at 1.13 kHz, (21±5 kPa) at 5.13 kHz, (28±2) kPa at 7.13 kHz and (30±3) kPa at 11.13 kHz with a statistic over five approach curves. The five curves for each excitation frequency are reported in figure 7.

The Force Modulation technique [33], applied to the study of the cell elasticity over the last years, typically has been looking at frequencies lower than 100 Hz [10], [34]. In this frequency range the cells show an increase of the Young modulus with frequency following specific power laws.

**Figure 8:**
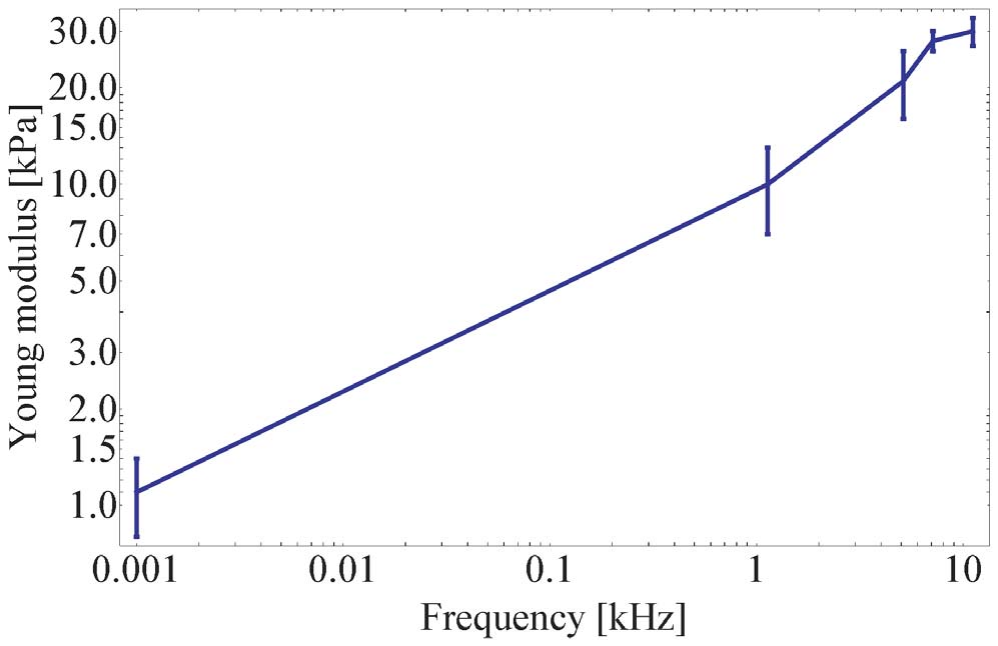
Frequency dependence of the PC12 Young's modulus.

In this work the study has been extended to frequencies larger than 1 kHz. At 1 kHz we observe a Young modulus which is one order of magnitude higher (figure 6) than in quasi-static condition (figure 3), which is coherent with what has been previously measured on living cells [10], [34]. The results are shown in figure 8. As reported in the previous section, no consistency has been found on the increase of the cell stiffness, which doesn't allow us for the moment to derive any specific power law in the 1–10 kHz frequency range. However, the observed cell elasticity in all the measurements performed in this frequency range has always been found in the order of ten(s) of kPa which is consistently higher than the one measured in quasi-static conditions.

While this paper was under review, a related article was published [35]. The authors have compared the quasi-static stiffness and the dynamic stiffness in the kHz range of the nuclear region of a live fibroblast cell. For large indentation depth, they observed an increase of the stiffness with the frequency which is consistent with our measurements.

## Conclusions

In conclusion, the newly introduced Force Feedback Microscope measures simultaneously the force, the force gradient and the damping factor at arbitrary excitation frequencies, fully characterizing the interaction between the AFM tip and the sample. In the present work we developed two spectroscopic protocols to study with the FFM the mechanical properties of living cells as a function of the frequency. The protocols have been applied to the study of PC12 living cells. At first, cells have been characterized with images at different constant forces and at different excitation frequencies. Then, the response of the cell membrane has been characterized through the acquisition of approach curves at different frequencies. The local mechanical impedance, that is the elasticity and the damping factor, have been measured and characterized in the 1 kHz–10 kHz range. Our measurements have been compared with data existing in literature revealing that the amplitude of oscillation applied on the tip is a crucial parameter when the cell elasticity and dissipation have to be characterized. The local mechanical impedance of the PC12 living cell has been characterized in the XY plane simultaneously to the cell morphology in analogy with already existing technique such as multi-harmonics AFM [16].

One of the future objectives is the extension of the lower and upper limits of the available excitation frequencies, below 1 kHz and above 15 kHz respectively.

## Supporting Information

Figure S1 **Force vs indentation curves.** These curves have been used in the statistics shown in the inset of Figure 3 of the manuscript. The statistics has been used to evaluate the value of PC12 Young's modulus. Blue: raw data. Red: fit using equation (3) in the manuscript.
(TIF)

Figure S2
**Five indentations curves for each excitation frequency.** a) Force gradient as a function of the tip indentation. a) 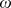  = 1.13 kHz, b) 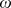  = 5.13 kHz, c) 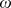  = 7.13 kHz, d) 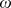  = 11.13 kHz. The lines in orange are the experimental linear fit of the cell elasticity for one of the five curve.(TIF)

Figure S3 **Amplitude (left) and phase (right) of the tip oscillation during an indentation experiment.** The indentation in blue is performed four times faster than the one in red. Clearly, slower experiments are less affected by noise.
(TIF)
