# Supplementary figures and images for: Spectroscopic Investigation of Local Mechanical Impedance of Living Cells
Source: PLoS One. 2014 Jul 7;9(7):e101687. doi: 10.1371/journal.pone.0101687 (PMC4084948; doi:10.1371/journal.pone.0101687)

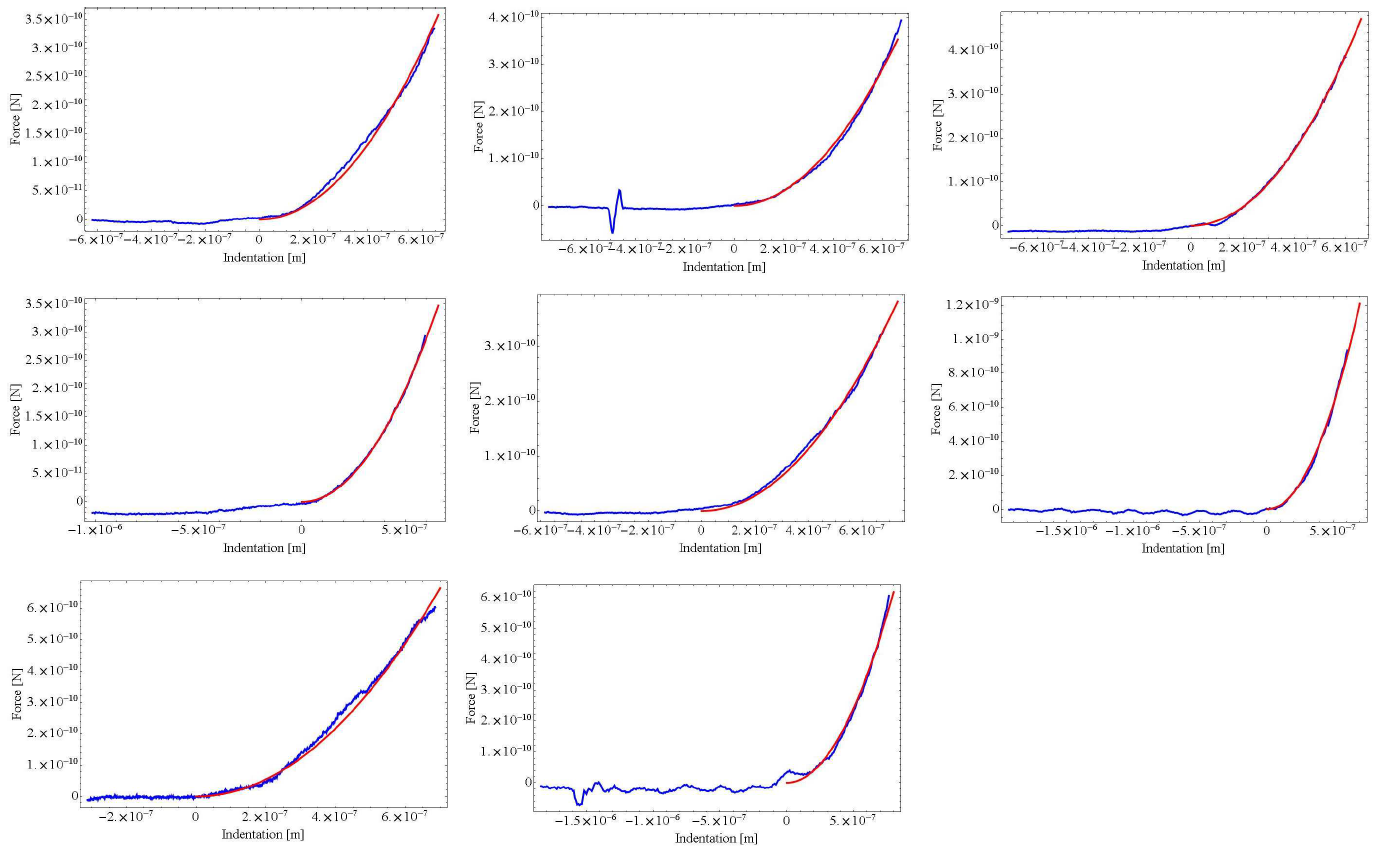

Supplement: Figure S1 — Force vs indentation curves. These curves have been used in the statistics shown in the inset of Figure 3 of the manuscript. The statistics has been used to evaluate the value of PC12 Young's modulus. Blue: raw data. Red: fit using equation (3) in the manuscript. (TIF) [file pone.0101687.s001.tif]

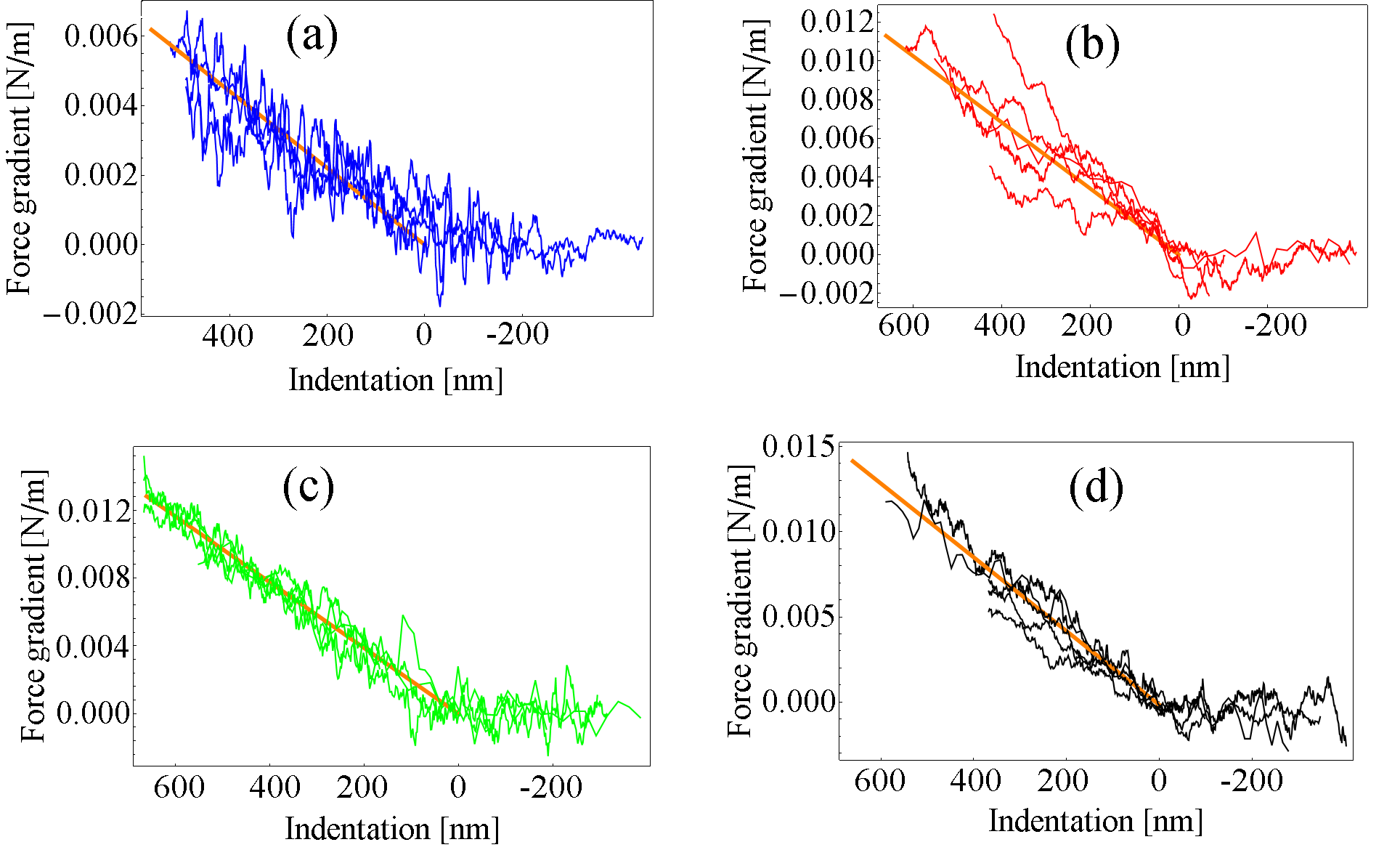

Supplement: Figure S2 — Five indentations curves for each excitation frequency. a) Force gradient as a function of the tip indentation. a) = 1.13 kHz, b) = 5.13 kHz, c) = 7.13 kHz, d) = 11.13 kHz. The lines in orange are the experimental linear fit of the cell elasticity for one of the five curve. (TIF) [file pone.0101687.s002.tif]

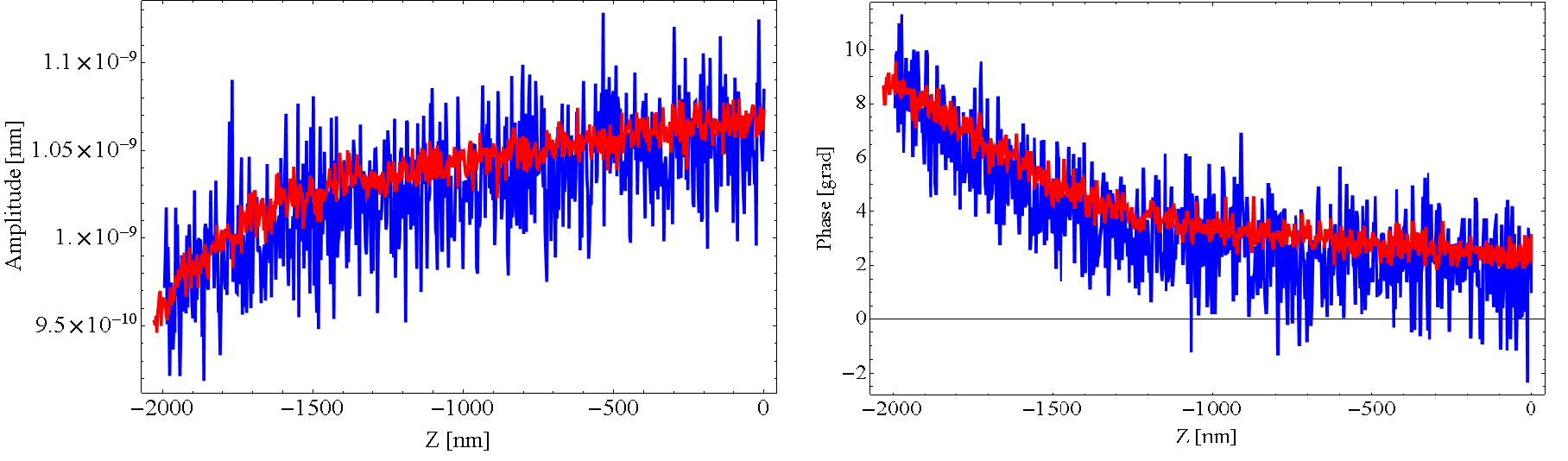

Supplement: Figure S3 — Amplitude (left) and phase (right) of the tip oscillation during an indentation experiment. The indentation in blue is performed four times faster than the one in red. Clearly, slower experiments are less affected by noise. (TIF) [file pone.0101687.s003.tif]
